# Treatment of Acute Articular Rheumatism by Veratrine

**Published:** 1853-09

**Authors:** 


### *Treatment of Acute Articular Rheumatism by Veratrine.*

By M. Trousseau.

We have already spoken of this treatment, but consider it of so much importance as to refer to it again. One pill, we have said, containing the tenth of a grain of veratrine, is given the first day, two the second, three the third, increasing the dose one pill each day, and it is rarely necessary to go beyond six or seven. M. Trousseau, knowing the duties of physician and teacher, has not hesitated to experiment with this therapeutical agent before his numerous students; the results have been marvelous. A woman, aged twenty-five years, entered his wards with a general acute articular rheumatism of three days' duration, complicated with endocarditis characterized by a souffle, to the first beat of the heart, extending itself into the calibre of the aorta, and into the arteries of the neck, plainly indicating a lesion of the aortic orifice. It was impossible to find a case more favorable for the therapeutic experiment on account of the extent and intensity of the complications. M. Trousseau gave a pill on Saturday; on Sunday there was already an amelioration of the symptoms, and he gave two pills. Monday, the fever, the inflammatory engorgement of the hands, &c., had almost disappeared: three pills were given. Tuesday, the day upon which M. Trousseau lectured upon the case, the health was perfect, except the condition of the heart. "A fact is but a fact," remarked the learned professor, "but I can hardly attribute this result to chance." Neither can we attribute it to chance, because we have seen M. Piėdagnel employ this medicine with a success almost constant; and know many cases which prove its efficacy. The innocence, the insipidity, and the low price of veratrine, will make it, (if farther experience confirms the facts already noticed,) one of the most precious medicines which we possess. We must not pause upon so good a road. What is rheumatism, if not an inflammatory affection, associated with the rheumatismal diathesis? And on the other hand, in a number of cases, what is pleurisy, pneumonia itself, etc., if not inflammatory conditions produced by the same general influence, rheumatism? It is true that the erratic march of the inflammation establishes a sensible difference between acute articular rheumatism, and pleurisy, and pneumonia, but if the principle is the same is there not a reason for trying the same remedy. We reason with reference to the specific antirheumatismal power which veratrine seems to possess. But if this is not satisfactory it may be regarded in another sense, viz., as a powerful antiphlogistic or contra-stimulant, and as such veratrine would be indicated in a general manner against inflammation, since it has been seen in the space of three days, in the dose of six-tenths of a grain altogether, to reduce the pulse. Perhaps we are indulging in too great anticipations with regard to the results of experiments with veratrine, and it will be necessary, at least at first, to limit its application to inflammatory affections, allied to rheumatism; among which we include pleurisy, pneumonia and neuralgia. The aortic souffle persisted in M. Trousseau's patient, but this is often the case with any treatment, and M. Piėdagnel asserts that there is a notable diminution in the diseases of the heart consequent upon acute articular rheumatism when it is treated with this medicine.—*Gazette des Hopitaux*, from *Virginia Medical and Surgical Journal.*

